# Foreseeing fates: a commentary on Manton (1928) ‘On the embryology of a mysid crustacean, *Hemimysis lamornae*’

**DOI:** 10.1098/rstb.2014.0381

**Published:** 2015-04-19

**Authors:** Michael Akam

**Affiliations:** Department of Zoology, University of Cambridge, Downing Street, Cambridge CB2 3EJ, UK

**Keywords:** malacostracan embryology, arthropod, development, evolution

## Abstract

Sidnie Manton became best known for her work on arthropod locomotion, and for proposing radical views on the evolution of arthropods that were accepted for a generation. However, her early training was as an embryologist, and the work that she carried out at the beginning of her career still stands as one of the major twentieth century contributions to the study of crustacean embryology. Here, I review her first major paper, largely completed while she was a graduate student, describing embryonic development in *Hemimysis lamornae*, a small shrimp-like animal found in the seas around the UK. The clarity of her writing and the quality of her figures set a standard that laid the basis for subsequent work, and although not all of her conclusions have stood the test of time, they remain a standard reference for work today. This commentary was written to celebrate the 350th anniversary of the journal *Philosophical Transactions of the Royal Society*.

Sidnie Manton is a towering figure in the twentieth century history of arthropod biology, as the leading and most forceful proponent of the belief that arthropods have evolved several times, independently, from worm-like ancestors. These views were based very largely on a series of studies on the functional morphology of different arthropod groups, and it is for this work that she is best known. However, as this paper shows, her first claim to fame was as an embryologist.

In 1927, when this paper, ‘On the embryology of a mysid crustacean, *Hemimysis lamornae*’ [1], was submitted, Sidnie Manton was 25 and had yet to obtain her PhD. She had graduated from Cambridge two years previously, coming top of the final year class list in Zoology (but of course, not receiving a full degree or being awarded the University prize that would normally go with this achievement: women could sit the courses and take the exams, but they would not be eligible to be awarded a bachelor's degree by Cambridge until 1947!). She had spent a year working as a graduate student at Imperial College with H. Graham Cannon, before returning to Cambridge as a demonstrator, the lowest academic post, in 1926 [2]. This paper therefore represents her first significant independent publication. In that light, it is truly remarkable, representing one of the most significant works of descriptive arthropod embryology to be published in the twentieth century. In a major synopsis of comparative embryology across the animal kingdom, published in 1997 [3], Scott Gilbert selected just two papers on crustaceans to summarize in detail—this paper from Manton, and a 1969 paper from Donald. T. Anderson, on barnacle embryology [4].

It is clear from her introduction that Manton began this work to clarify questions about the later development of the coelomic cavities, the heart and the excretory organs in malacostracan Crustacea, questions which arose from the earlier work of her supervisor, Cannon, on the same organs in other crustaceans. Already, when she began, there were many descriptions of early development in Malacostraca—for this group includes the large and economically important crustaceans that we eat, the crabs, lobsters and shrimps, as well as woodlice, mysids and many other less familiar forms. However, Manton quickly concluded that most of these existing descriptions were, to use her own words, ‘incomplete, and often inaccurate’ (p. 364). ‘It became evident that an examination of the earliest stages was equally necessary’ (p. 364), she wrote, and her work extended to cover the whole period of development, from the youngest available eggs through to the hatching larva.

Her paper, running to just over 100 printed pages, continued a tradition of comparative embryology at Cambridge that dated from the nineteenth century. Francis Maitland Balfour had established the Morphological Laboratory in the late 1870s, making Cambridge at least briefly a major international centre for evolutionary and comparative embryology [5]. Workers like Adam Sedgwick and Sidney Harmer were then describing the development of hitherto little studied organisms such as velvet worms and bryozoans, and establishing a modus operandi that came to be time-honoured. Embryos representing all stages of the animal's development were pickled (fixed is the technical term), and embedded in wax or a similar medium that allowed them to be sliced into sections a few thousandths of a millimetre thick. These sections were then laid out on slides, such that each embryo was represented by a series of sections from top to bottom.

It was then the job of the embryologist to examine, painstakingly, the series of sections of each timepoint, to build in their mind (or indeed in wax) a three-dimensional model of the embryo, and then to compare these across timepoints to infer the four-dimensional sequence of development through time, representing the movements of cells and transformations of tissues that convert a single-celled ovum into a complex animal.

This same task is still at the heart of much developmental biology, but we have expensive new toys that allow the images to be collected from living embryos, so that the timecourse of development can be played back literally as a movie. Fluorescent reporter proteins encoded by transgenes make the nucleus of every cell visible; techniques such as light-sheet microscopy collect serial sections at each timepoint by sweeping a thin plane of light through the embryo, allowing the whole embryo to be visualized in three dimensions at cellular resolution ([6,7]; figure 1). With the benefit of hindsight, these techniques have shown us that inferring what is actually happening from a series of stills is very difficult, and even the most careful investigator makes mistakes!

**Figure 1.**
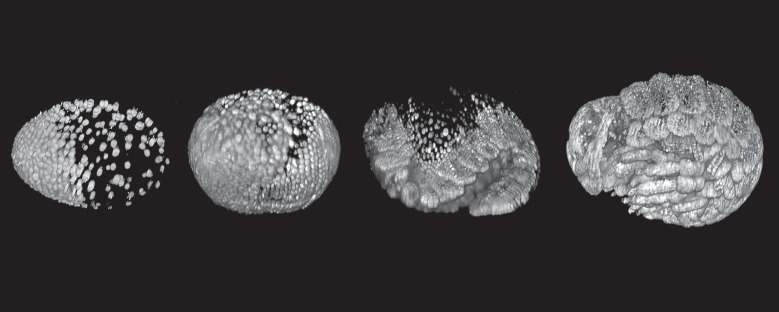
Following crustacean development in a live embryo at single-cell resolution with modern techniques. The figure shows four time points during the development of an embryo of the amphipod crustacean *Parhyale hawaiensis*, imaged using multi-view fluorescence light-sheet microscopy (lateral views, anterior to the left). The nuclei are fluorescently labelled using a transgenic construct. The image at the left shows an early stage while cleavage nuclei are aggregating to form the embryonic primordium; the image at the right shows a late differentiation stage, with the forming antennae and limbs clearly visible. A full movie of *Parhyale* development is available at the link http://www.cell.com/pictureshow/lightsheet2, which also explains further how the data were collected. Images courtesy of Anastasios Pavlopoulos.

But when Sidnie Manton set out on this project, she was using the very best techniques of the day. Indeed, one of the reasons why crustacean embryology had been so refractory to progress was that the large yolky eggs of many crustaceans were extremely difficult to fix in such a way that they could be sectioned without distortion or loss of material. In her paper, she emphasizes that ‘all previous work on yolky eggs had been based on sublimate or worse fixatives’ (p. 364), and frequently throughout her discussion she comments that previous observers were in error because of the methods used. Graham Cannon suggested that she use ‘B.G. Smith's fluid’, previously developed for fixing amphibian yolk, and this, together with improved embedding and staining methods, proved to be the magic trick required to procure excellent serial sections, so important for preserving the fine layering of tissues that allowed the topology of the embryo to be reconstructed.

But, one suspects, it was not just the improved techniques that made this work such a landmark. Sidnie Manton was a remarkable artist, who since childhood had been drawing and painting natural history specimens with great accuracy [2]. Five plates of her drawings accompany this article, depicting not just the cells and tissue layers in each section, but also the distinct appearance of the nucleus within each cell, details that allowed her to discriminate with great confidence endoderm from ectoderm, germ cell from mesoderm (figure 2). Today, we would require molecular markers to make these calls, but for her contemporaries, who had spent many long hours studying just such preparations, these meticulous plates must have been highly persuasive.

**Figure 2.**
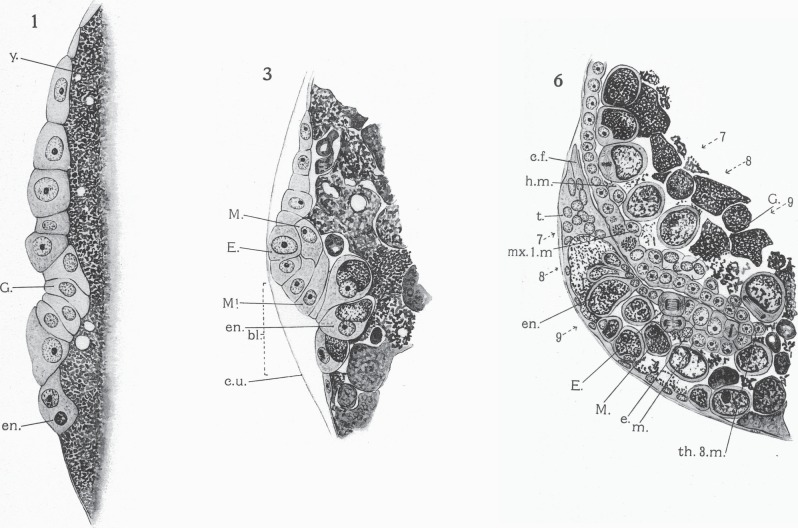
Sections of fixed and stained embryos of *Hemimysis*, as figured in the plates accompanying Manton's paper [1], showing her attribution of cell identities. Plate 1: The beginning of gastrulation. A single layer of cells overlies the yolk (y). The future germ line cells (G) are starting to buckle inwards at the blastopore. Plate 3: A later stage in gastrulation, with ectodermal teloblast (E), mesodermal teloblast (M) and endoderm (en) all identified in the region of the blastopore (bl). Plate 6: A later stage, after the caudal end (c.f.) of the embryo has flexed forward, showing the rich detail of cell differentiation in these preparations. Copyright © The Royal Society.

The plates are complemented by text figures, somewhat schematized, which are not just diagrams but encapsulations of her understanding (figure 3). Using arrows to show inferred cell movements, and in some diagrams figuring internal tissues in red and superficial tissues in black, she illustrated with remarkable clarity the origin and relations of the different germ layers, and the complex tissue movements that generate the body plan of this shrimp.

**Figure 3.**
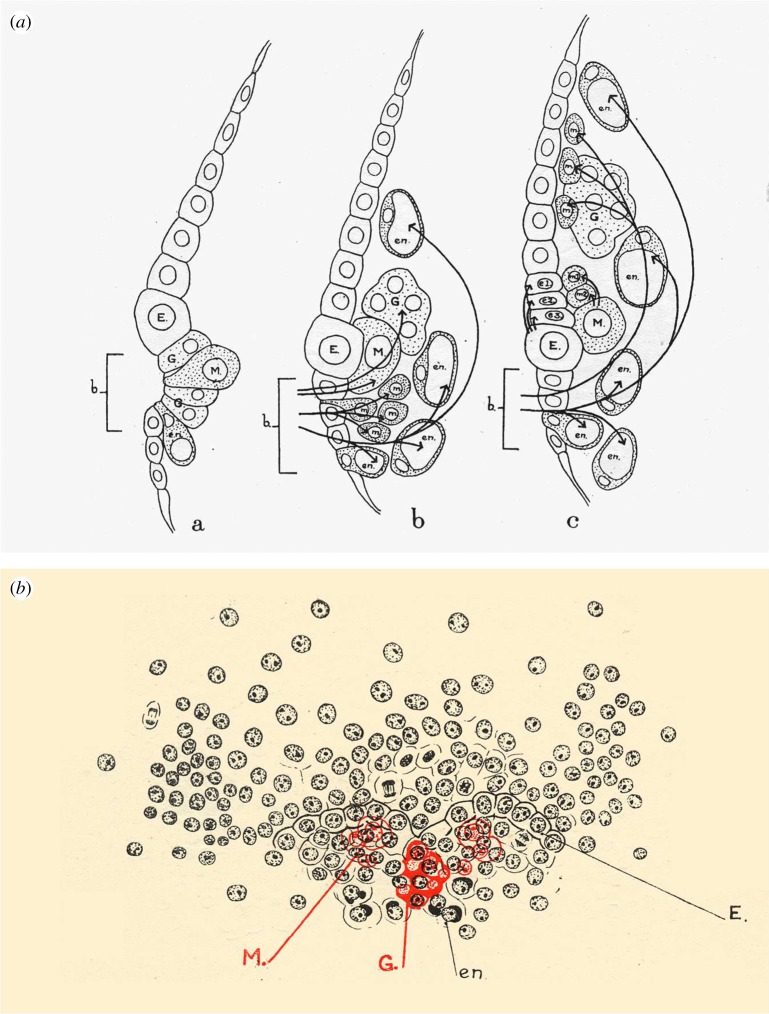
Figures from Manton's paper [1]. (*a*) Fig. 3a–c: sections through the blastoporal area (b), showing inferred cell fates and movements during three stages of gastrulation. (*b*) Fig. 5a: a surface view of a stage between fig. 3a and b, showing Manton's use of colour to depict internal cells in red (mesodermal teloblasts and germ line precursors) beneath the surface cells in black. M, head mesoderm; e1, e2, e3, descendants from ectodermal teloblasts; m1, m2, descendants from mesodermal teloblasts. See the original paper for all other abbreviations. Copyright © The Royal Society.

To a modern reader, the confidence that she brings to her interpretation is breathtaking. She writes as though she has seen the movie and is describing the process of embryology after running it forwards and backwards before her eyes, as we could today: ‘The primordium slips into the interior’ (p. 376); ‘a wave of division seems to pass from the middle line outwards, the inner teloblasts dividing a little in advance of the outer’ (p. 371). Sometimes one can see exactly how she made these inferences, about the cell divisions for example, but for some other claims I wonder how she could possibly have known with such confidence. Modern referees would not be satisfied; they would ask that cells be marked, that lineages be traced, that the figures actually prove statements which here we have to take on trust.

Were all her interpretations correct? In many cases, we still do not know, because no equally thorough work has since been done on this species, and certainly none using the fanciest modern microscopy that would test her hypotheses most effectively. However, there are some conclusions which, from studies of closely related species, we would today regard as surprising and likely suspect (see below). She herself certainly did not trust many of those who had previously used similar techniques to study related embryos. Referring to an earlier paper on a different species of mysid, she wrote

However, with the general inaccuracy which characterises this work, he considers it to arise by … He clearly did not distinguish between … nor did he follow carefully the formation of … (p. 439).

Not a lady to mince her words, Sidnie Manton! Some earlier workers she does respect—but even the best embryologists, using these classical techniques, made mistakes. In our own work, we have found that one classic paper by Richard Heymons, which provided the best description of centipede embryology for a hundred years, muddled up the future mouth and anus at one point in development [8].

Foreshadowing Manton's later work, this paper goes beyond the usual territory of comparative embryology, to deal in great detail not just with the origins of tissues and organs, but also with the subsequent development of the musculature and tendons, mapping in detail their attachments in each segment (figure 4). She is effectively laying the foundations for functional morphology.

**Figure 4.**
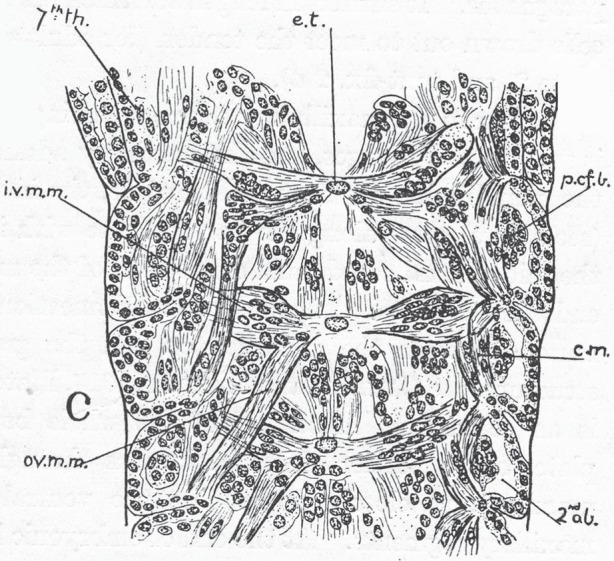
Fig. 23 in Manton's paper [1], of a much later embryo, showing detail of the differentiation of the tendon and muscle system in the posterior thorax (7^th^ th) and anterior abdomen (2^nd^ ab). e.t, ectodermal tendon rudiment; ivmm, ovmm, c.m., different muscles. Copyright © The Royal Society.

For the nineteenth and early twentieth century comparative embryologists, the objective of all this meticulous work was not just to generate a description of the species, but, by interpreting this description in the context of what was known from other species, to reveal the transformations that have generated modern forms from their ancestors.

In this context, the subject of study, *Hemimysis,* was carefully chosen. Although mysid shrimps are malacostracan or ‘higher’ crustaceans, Manton considered them to represent one of the least specialized forms, with a morphology that was relatively easy to relate to the inferred ancestral state of the Malacostraca. Mysids do not develop a free swimming nauplius larva—a larval form that is inferred to be ancestral for the crustaceans as a whole—but the development of the region in the embryo corresponding to the segments of the nauplius is clearly distinct from the subsequent development of the segments of the trunk, which are mostly added through a series of teloblast (stem cell) divisions that generates a regular lattice of segment primordia.

This much had long been known when Sidnie Manton started her work [9], but she added key observations to the description. For example, she inferred the existence of a seventh abdominal segment in the embryo, even though no such segment is distinguishable in the animal after hatching: most malacostracans have six abdominal (pleonic) segments. She observed that an additional row of cells is generated in the lattice of teloblast-derived segment precursors, after that for the sixth abdominal segment. The descendants of these cells later fuse with the sixth segment. This seventh segment would make the segmentation of *Hemimysis* directly comparable with that observed in leptostracan crustaceans like *Nebalia*, which have seven abdominal segments, supporting the idea that this was the ancestral condition among malacostracans. It had long been proposed that leptostracans occupied a position intermediate between the Malacostraca proper and the ‘lower’ crustacean forms then placed in a taxon called the Entomostraca, and Manton's observation fitted that model well.

One of the longest running arguments in arthropod biology concerns the structure of the arthropod head. Is there a territory at the anterior of the arthropod embryo that is, in evolutionary terms, derived from an asegmental territory—an acron—or are all the structures of the head ancestrally derived from modified segments, and if so how many are there? Manton concluded that the head musculature of *Hemimysis* had two quite different origins. She observed a localized ingression of cells in the anterior part of the head, which she thought gave rise to the most anterior head muscles. Muscles in the more posterior part of the head derived from cells ingressing more posteriorly, through the blastopore, and subsequently moving forward. She interpreted this as supporting a pre-antennulary segment in the head, rather than as supporting an a-segmental origin of the anterior head.

In this and other sections of the paper, she distinguishes very clearly between those characteristics that she believes to be primitive for the group (i.e. similar to those in the last common ancestor of the group) and those which she believes to be derived, representing states that have evolved within Crustacea, somewhere along the specific lineage leading to *Hemimysis*. She is quite clear that the formation of trunk mesoderm by teloblasts for example is a derived characteristic of the Malacostraca, and not directly comparable with a similar process that is observed in some annelids (e.g. leeches), whereas the formation of posterior head mesoderm by anterior migration from the blastopore she believes to be a primitive characteristic of crustaceans, and possibly of the arthropods as a whole. She asserts that her discovery of the origin of the most anterior head mesoderm from a local invagination within the head lobe is also a derived characteristic, though believes that it exists in many other Malacostraca previously studied, where it had not been recognized. Why she favoured this interpretation is not clear to me. Indeed, the details of head segmentation and head mesoderm formation remain unclear today: they were perhaps simply too complex to be resolved by the techniques then available.

This paper on *Hemimysis* was not Sidnie Manton's last contribution to crustacean embryology. Six years later, she submitted a second paper [10], of similar extent, on the embryology of the leptostracan *Nebalia bipes*, that basal malacostracan with which it had been germane to compare *Hemimysis*. This subsequent paper follows a similar structure and pattern to her earlier work, though the plates in the later paper are more stylistic and less artistic. Perhaps pressures on her time were now greater, or perhaps fashions had just changed. The key finding of this paper was that the embryology of *Nebalia* was in almost all respects comparable with that of other malacostracans. She concluded that its embryology ‘gives no assistance in any attempt to bridge the gulf between the higher Crustacea and the other groups’ [10, p. 225].

There was one more major work of embryology to come, in 1949, on velvet worms (onychophorans), published like the others in *Philosophical Transactions* [11]. However, that was to be Manton's last major endeavour as an embryologist.

Right at the outset of her career, she had begun, with Cannon, to work on the feeding mechanisms of mysid shrimps and other crustaceans [12,13]. In 1928, she carried out extensive fieldwork in Tasmania and Queensland, some of it with the Great Barrier Reef expedition led by Sir Maurice Yonge. Following on from this, she published a number of field-based studies. During the 1940s, she turned her attention to velvet worms, completing a series of papers on many different aspects of their biology—feeding, water relations, taxonomy, life history and fertilization biology, as well as embryology.

The last of these works on velvet worms, ‘The locomotion of *Peripatus*’ [14] published in 1950, was also the first in what was to become her most famous series of papers—*The Evolution of Arthropodan Locomotory Mechanisms.* Over the next 23 years, further studies appeared on the locomotion of centipedes and millipedes, of hexapods and others. The series ran to 11 parts, and over 900 pages, defining a field. It culminated in a major book, *The Arthropoda: Habits, Functional Morphology and Evolution* [15], written shortly before her death. During this time, she published occasional brief papers on embryology (e.g. on pogonophorans [16]). But, after the second crustacean paper, she appears to have decided that, at least for her own work, descriptive embryology was not the main thrust: functional studies were the thing.

Despite this, she developed her ideas on arthropod evolution throughout her career in close collaboration with two other comparative embryologists who took the work forward, even when it had become unfashionable. Both were in Australia—Oscar W. Tiegs in Melbourne, publishing on myriapod embryology throughout the 1940s, and later Donald T. Anderson in Sydney, working mostly with crustaceans and insects. These two continued in very much the same tradition, using essentially the same embryological techniques. They published chunky monographs, Anderson in *Philosophical Transactions* (e.g. [4]), Tiegs (e.g. [17,18]) in the *Quarterly Journal of Microscopical Science* (the forerunner of today's *Journal of Cell Science*). They would develop the thesis, argued at length by Anderson in his book *Embryology and Phylogeny in Annelids and Arthropods* [19], that embryology, and particularly the early fate maps of the embryos, provided independent support for the polyphyletic origin of arthropods, a view that Sidnie Manton's studies of functional morphology had by then led her to espouse with great conviction. Both Manton and Anderson argued that onychophorans, myriapods and insects formed one phylum of arthropods, the Uniramia, to the exclusion of crustaceans and chelicerates. They believed that other arthropods had become ‘arthropodized’—that is, encased in a hardened cuticle and possessing jointed limbs—quite separately, evolving from different worm-like ancestors. In the final chapter of her 1977 book, she wrote ‘… comparative functional embryology … points clearly to the independent evolution of (1) the Uniramia … (2) the Crustacea … (3) the Chelicerata’ [15, pp. 487–488]. This view dominated at least the English speaking world for more than 20 years.

The 1928 *Philosophical Transactions* paper only hints at these later ideas—but in Manton's figuring of the presumptive areas of early crustacean embryos (her text, fig. 29), and her discussion of this in the context of relationships within the Crustacea, we see the first clear statement of the idea that was to be built on by Anderson, and which so strongly influenced Manton later.

How has time treated these ideas? It is probably fair to say that Manton's papers, together with those of Anderson, Tiegs and others working in the 1950s and 1960s, represented a last major flowering of purely descriptive arthropod embryology: nothing of quite the same scope could be published now. In the modern era, the claims that Manton could make *ex cathedra* have to be proved by experimental methods of cell marking and lineage tracing, and whole series of beautiful papers have been published verifying or refuting statements that would occupy just a short paragraph in Manton's work—on the origins of the mesoderm, for example, or the relation of segmental organization to teloblastic cell divisions.

Over the last 40 years, this experimental tradition of crustacean embryology has been developed most actively in Germany (e.g. by Wolfgang Dohle, Gerhard Scholtz and colleagues in Berlin [20–22]). In recent years, it has received a new impetus with the development of *Parhyale hawaiensis* as a new laboratory model organism, pioneered by Bill Browne and Nipam Patel at Berkeley [23] and now being adopted in a number of laboratories worldwide [24–26] (figure 1). But it is worth noting that Dohle wrote [27] in 1986

When I switched to investigating crustacean embryology, it was the work of Sidnie Manton (1928, 1934) that gave me most inspiration (p. 1).

The clarity of her description, presented in relation to then current questions, and in the context of a critical appraisal of all past work, provided the basis on which crustacean embryology moved forward [27].

Besides experimental papers, there are still descriptive papers on specific species, particularly those of economic interest, but most of these bear no comparison with Manton's detailed work, or indeed with the major monographs on crustacean embryology published in the late nineteenth and early twentieth centuries [9,28] when comparative embryology was the height of fashionable science. Such studies have not been high profile science for many years and would not typically get into the pages of *Philosophical Transactions* or other broad interest journals. Papers on Crustacea in those journals are now more likely to be discussing Cambrian fossils or the genetic control of body plans than descriptive embryology. Hence, it is hardly surprising that Gilbert and Raunio were still able to write in 1997 ‘While crustaceans are among the most widespread species on earth, we know very little about their embryology. There are very few studies following the development of any one species in detail’ [3, p. 238]. The same remains true today.

No subsequent detailed experimental studies have been carried out in *Hemimysis lamornae*, so some of the more surprising conclusions of Manton's work remain to be validated. One area where her work is likely in error is in the precise parts of the body deriving from the stem cells of the teloblastic rows. She put the anterior boundary of teloblast-derived tissue at the posterior of the mandibular segment, whereas lineage tracings in other malacostracans show this boundary to lie posterior to the second maxillary segment, and this is true also for mysids [21]. Also, the idea that the extensor and flexor muscles of the limbs in mysids have an ectodermal origin is not consistent with lineage studies in other malacostracans, which show that all muscles derive from an early specified mesoderm lineage and that even during regeneration there is no compensation between lineages [29–31]. Whether or not this is also true for *Hemimysis* remains to be proved.

In terms of the larger picture, the fate of Manton's ideas has been mixed. Her views on the homology of arthropod head segments are consistent with those supported by Hox gene data, and now widely accepted—that the antennal segment of insects and crustaceans is homologous to the cheliceral segment of chelicerates and that both are preceded by a segment without appendages (ocular/preantennal/precheliceral), which may be homologous to the antennal segment of onychophorans [32].

However, her views on arthropod polyphyly, absolutely fundamental to her whole picture of arthropod evolution, are not consistent with the current consensus. In particular, the concept of a uniramian clade uniting onychophorans, myriapods and hexapods to the exclusion of crustaceans and chelicerates is clearly wrong, if molecular phylogenetics has any validity at all. The consensus now overwhelmingly supported by molecular data, including major phylogenomic studies using thousands of genes [33–35], is that living arthropods as conventionally defined (i.e. insects, crustaceans, myriapods and chelicerates) do represent a monophyletic clade. Onychophorans are the likely sister group to this whole clade.

Where does that leave comparative embryology, and indeed functional morphology, as phylogenetic tools? Are they fundamentally flawed, or was it simply their application in this context that was faulty? Manton recognized, quite correctly, that

any view of arthropod phylogeny, monophyletic or otherwise, commits us to a good deal of convergence—the independent evolution of fairly similar structures, such as tracheae, compound eyes, plastron respiratory structures, the shapes and enrolling ability of pill millipedes and of certain isopods and many other examples [15, p. 237].

Yet she was absolutely convinced of the quality of her data

This evidence is not speculative, it is sound; and the broad conclusions which have been reached [by functional morphology] tally with and are substantiated by the recent work on comparative functional embryology of annelids and arthropods, the latter using the illuminative concepts ‘fate maps’ of presumptive areas of the blastula … This is the stage at which the fundamental framework of the body is established. The study of functional morphology of arthropods over certain fields has yielded more detailed evidence than any embryology can be expected to provide, but in other fields the embryological evidence is more compelling. It is most significant, and satisfactory that the two lines of modern work are in such close agreement [15, p. 237].

For me, there is a disconnect here in Manton's thinking. She is adamant that simplified diagrams have no place in functional morphology

No proper understanding of either function or morphology can be obtained from simplified diagrams, which thus become a menace and a hindrance [15, p. 491].

And yet, when it comes to embryology, she put great faith in the diagrammatic fate maps that appeared to unite the uniramian groups. Was this perhaps, because she overestimated the fixity of the earliest stages in embryology—believing that these early stages could not and had not diverged in response to selective pressures? It seems surprising, but why else would she have been so enamoured of these overly simplistic diagrams? With the benefit of hindsight, it seems that, in the context of both embryology and functional morphology, she underestimated the extent to which even the most fundamental biological processes could be modified by evolution.

And yet, the detailed descriptions, both of embryology and of functional morphology, stand as a lasting testament to her experimental skill, her artistic talents and to the clarity of her thinking, which shines through in her writing. She carried a whole generation with her, including not just observers of her science but those best qualified to assess and criticize, embryologists like Don Anderson and functional morphologists like Geoffrey Fryer, some of whom to this day remain loyal to the Uniramia, and doubt the validity of techniques that suggest other possibilities. Perhaps the last word in this debate is not yet spoken.

## Author profile

**Michael Akam** is an embryologist and geneticist with an interest in the evolution of developmental mechanisms. He participated in the first isolation of the developmental control genes known as Hox genes and has since worked on many aspects of segment formation and segment patterning in insects and other arthropods. Over the last 20 years, he has exploited our growing knowledge of developmental genetics to study the genetic basis for animal diversity, working most recently with centipedes. In 1989, he moved as a founding member to the Wellcome/CRC Institute for Cancer and Developmental Biology in Cambridge. In 1997, he became Director of the University Museum of Zoology, and in 2010, Professor of Zoology and Head of the Department of Zoology. He is a Fellow of Darwin College.
